# Ubiquitin-Specific Proteases 25 Negatively Regulates Virus-Induced Type I Interferon Signaling

**DOI:** 10.1371/journal.pone.0080976

**Published:** 2013-11-18

**Authors:** Huijuan Zhong, Dang Wang, Liurong Fang, Huan Zhang, Rui Luo, Min Shang, Chao Ouyang, Haiping Ouyang, Huanchun Chen, Shaobo Xiao

**Affiliations:** State Key Laboratory of Agricultural Microbiology, College of Veterinary Medicine, Huazhong Agricultural University, Wuhan, China; Temple University School of Medicine, United States of America

## Abstract

Ubiquitination and deubiquitination have emerged as critical regulatory processes in the virus-triggered type I interferon (IFN) induction pathway. In this study, we carried out a targeted siRNA screen of 54 ubiquitin-specific proteases (USPs) and identified USP25 as a negative regulator of the virus-triggered type I IFN signaling pathway. Overexpression of USP25 inhibited virus-induced activation of IFN-β, interferon regulation factor 3 (IRF3) and nuclear factor-kappa B (NF-κB), as well as the phosphorylation of IRF3 and NF-κB subunit p65. Furthermore, Knockdown of USP25 potentiated virus-induced induction of the IFN-β. In addition, detailed analysis demonstrated that USP25 cleaved lysine 48- and lysine 63-linked polyubiquitin chains *in vitro* and *in vivo*, and its deubiquitinating enzyme (DUB) activity, were dependent on a cysteine residue (Cys178) and a histidine residue (His607). USP25 mutants lacking DUB activity lost the ability to block virus-induced type I IFN to some degree. Mechanistically, USP25 deubiquitinated retinoic acid-inducible gene I (RIG-I), tumornecrosis factor (TNF) receptor-associated factor 2 (TRAF2), and TRAF6 to inhibit RIG-I-like receptor-mediated IFN signaling. Our findings suggest that USP25 is a novel DUB negatively regulating virus-induced type I IFN production.

## Introduction

Innate immune responses are activated through host pattern recognition receptors (PRRs), which recognize molecular structures called pathogen-associated molecular patterns (PAMPs) that are structurally conserved within large groups of pathogens. Upon engagement of PAMPs, PRRs initiate signaling pathways, ultimately triggering the production of type I interferons (IFNs) [1]. The type I IFN signal is then transducted by the activation of Janus kinase (JAK) family protein kinases and the signal transducers and activators of transcription (STAT) family of proteins [2], resulting in the generation of the transcriptional activator, IFN-stimulated gene factor 3 (ISGF3). ISGF3 bind to their specific DNA sequences containing each common motif; namely, the IFN-stimulated response element (ISRE), and participates in IFN responses. IFN stimulation of promoters containing ISRE results in the transcriptional induction of a large number of IFN-stimulated genes (ISGs) to evoke antiviral activity [3].

Studies during the past decades have revealed a working model on virus-triggered type I IFN signaling. Recognition of virus-derived double-stranded RNA (dsRNA) and 5’-triphosphorylated single-stranded RNA (5’pppssRNA) by retinoic acid-inducible gene I (RIG-I) and melanoma differentiation-associated gene 5 (MDA5) in the cytoplasm lead to the production of type I IFN and inflammatory cytokines [4-6]. RIG-I/MDA5 is indispensable for type I IFN responses to many RNA viruses, including influenza A virus, mumps virus, measles virus and Sendai virus (SEV) [7,8]. After sensing cytoplasmic viral RNAs, RIG-I and/or MDA5 interacts with the caspase activation and recruitment domain (CARD)-containing protein IFN-beta promoter stimulator 1 (IPS-1, also known as MAVS/VISA/Cardif) via CARD-like domains on both the RNA sensors and IPS-1 [9-12]. In turn, the IPS-1 complex recruits the downstream adaptor proteins tumornecrosis factor (TNF) receptor-associated factor 2 (TRAF2), TRAF3 and TRAF6, and TANK-binding kinase 1 (TBK1) and I kappaB kinse (IKK) α/β, leading to activation of the critical transcription factors interferon regulation factor 3 (IRF3) and nuclear factor-kappa B (NF-κB) [9-11]. Phosphorylated IRF3 and NF-κB translocate into the nucleus and directly induces type I IFNs, an array of cytokines and other mediators required for host defense [13,14].

Ubiquitination is a highly regulated process that has been found to be involved in a myriad of cellular functions [15,16] such as apoptosis [17], DNA repair, cell cycle regulation [18], proteasome-dependent protein degradation [19], kinase activation and signal transduction [20-22]. Multiple ubiquitin ligases and ubiquitin-binding scaffold proteins contribute to positive regulation of the antiviral innate immune response, such as RIG-I, TRAF2, TRAF6 and TBK1. Post-translational modification of proteins by the covalent ligation of ubiquitin is a crucial regulatory mechanism, and the enzymes that catalyze these modifications have been the focus of many studies [23]. Deubiquitinating enzymes (DUBs), which mediate the removal and processing of ubiquitin to recycle proteins, have a wide range of biological activity [24]. In recent years, the functions of DUBs in various pathways and signaling networks have been well reviewed. Accumulating evidence has revealed critical roles of deubiquitination in RIG-I-like receptor-mediated IFN signaling [25]. For example, deubiquitinating enzyme A (DUBA) selectively cleaves the lysine-63-linked polyubiquitin chains on TRAF3, resulting in its dissociation from the downstream signaling complex containing TANK-binding kinase 1 (TAK1) [20]; the tumor suppressor cylindromatosis (CYLD) removes lysine-63-linked polyubiquitin chains from RIG-I, which inhibits the IRF3 signaling pathway [26]; A20 removes lysine-63-linked polyubiquitin chains from receptor interacting protein (RIP) [27].

Despite the importance of DUBs, the functions of DUBs during the antiviral innate immune response are largely unknown. To study the ubiquitin-specific proteases (USPs), as a subclass of the DUBs superfamily, we synthesized a collection of siRNA to suppress 54 human USPs, and used these siRNA to identify the enzymes regulating the antiviral immune response. By siRNA screening, we identified a cellular deubiquitinase, USP25, which regulates the virus-induced ISRE promoter. We found that USP25 processes K48-linked and K63-linked polyubiquitin *in vitro* and *in vivo*. Through its DUB activity, USP25 cleaves ubiquitin moieties from critical signaling proteins of the type I IFN signaling pathway, RIG-I, TRAF2, and TRAF6. Our findings present evidence that USP25 functions as a deubiquitinase that significantly inhibited virus-induced type I IFN signaling pathway.

## Materials and Methods

### Expression plasmids and small interfering RNA

The plasmids pNF-κB-Luc and pISRE-Luc were from Stratagene. (PRDIII-I)_4_-Luc was kindly provided by S. Ludwig (Heinrich Heine University, Düsseldorf, Germany) [28]. Full-length hemagglutinin (HA)-tagged ubiquitin (Ub) mutants in which all but one Lys residue (HA-K48-Ub or HA-K63-Ub) was replaced with Arg were gifts from T. Ohta (St. Marianna University School of Medicine, Japan) [29]. pcDNA3.1-Flag-Ub and the IPS-1 expression vector were previously described [30,31]. The expression plasmids for wild-type (WT) RIG-I (pEF-Flag-RIG-I), its constitutively active mutant (pEF-Flag-RIG-I-N), and p125-Luc (IFN-β-Luc) were kindly provided by T. Fujita (Tokyo Metropolitan Institute of Medical Science, Tokyo, Japan) [32]. The TRAF6 expression vector was a gift from Edward W. Harhaj (University of Miami School of Medicine, Miami, FL, USA). The expression plasmid for TRAF2 was constructed by PCR amplification of cDNA of TRAF2 (GenBank Accession No. NM021138) from HEK-293T (human embryonic kidney epithelial) cells, followed by cloning into the pCMV-Tag 2B vector (Stratagene).

The hemagglutinin (HA) or Myc epitope tag was amplified by PCR and cloned into the pCAGGS-MCS [33] vector to generate a pCAGGS-HA or pCAGGS-Myc plasmid with N-terminally HA or Myc tag, respectively. For construction of pCAGGS-HA-USP25 or pCAGGS-Myc-USP25, the cDNA fragment encoding the full-length USP25 (GenBank accession no. NM013396) was amplified by PCR from cDNA of HEK-293T cells and subcloned into the pCAGGS-HA or pCAGGS-Myc vector, respectively. Mutagenesis of individual amino acid residues (C178A and H607A) in USP25 were conducted using overlap extension PCR. Detailed sequences of the specific primers used are available upon request. All constructs were validated by DNA sequencing.

Double-stranded oligonucleotides corresponding to the target sequences of 54 USPs were synthetized by Sigma-Aldrich. The target sequences (5’ to 3’) of USP25 used in this study are listed in Table S1. The siRNA sequences (5’ to 3’) of other USPs used are available upon request.

### Cell Culture and Virus

HEK-293T cells were cultured and maintained in RPMI-1640 (HyClone) supplemented with 10% heat-inactivated fetal bovine serum (FBS) at 37°C in a humidified 5% CO_2_ incubator. SEV was obtained from the Centre of Virus Resource and Information, Wuhan Institute of Virology, Chinese Academy of Sciences.

### Transfection and reporter assay

Transient transfection was performed using Lipofectamine 2000 (Invitrogen). HEK-293T cells grown in 24-well plates were co-transfected with 0.1 μg of luciferase reporter plasmid (p125-Luc for IFN-β, (PRDIII-I)_4_-Luc for IRF3, pNF-κB-Luc for NF-κB, and pISRE-Luc for ISRE), 0.02 μg of the Renilla luciferase construct phRL-TK (Promega) (for normalization of transfection efficiency) and various other expression plasmids or an empty control plasmid. In some experiments, cells were further infected or mock infected with SEV (10 hemagglutinating activity units/well) at 24 h after the initial co-transfection. Sixteen hours after SEV infection, cells were harvested. Luciferase assays were performed using a dual-specific luciferase assay kit (Promega) according to the manufacturer’s protocol. The relative luciferase activity was calculated by dividing the Firefly luciferase activity by the Renilla luciferase activity. All reporter assays were repeated at least three times. Data are presented as means ± standard deviation (SD).

### Reverse transcription-PCR

Total RNA was isolated from HEK-293T cells using TRIzol reagent (Invitrogen). One microgram of this total RNA was reverse transcribed to cDNA using avian myeloblastosis virus (AMV) reverse transcriptase (Toyobo, Japan), which (1 μL of 20 μL cDNA) was subsequently used in a SYBR green PCR assay (Applied Biosystems). The abundance of individual mRNA transcript in each sample was assayed three times and normalized to that of porcine glyceraldehyde-3-phosphate dehydrogenase (GAPDH) mRNA (as an internal control). The primers were designed with Primer Express software v.3.0 (Applied Biosystems). The sequences of the primers are listed in Table S2.

### *In vitro* deubiquitination assay

Wild-type HA-tagged USP25 protein was purified from cells transfected with pCAGGS-HA-USP25 using a HA tagged Protein PURIFICATION KIT (MBL) according to the manufacturer’s protocol. As a negative control, a HA tagged Protein PURIFICATION KIT was also used to isolate proteins from empty-vector transfected cells. Polyubiquitin chains were purchased from Boston Biochem (K48-Ub_2-7_ (Catalog No. UC-230) and K63-Ub_2-7_ (Catalog No. UC-330)). The purified products (2 μL) were incubated with 3.5 μL of K48-Ub_2-7_ chains or K63-Ub_2-7_ chains at 37°C in a 14.5 μL reaction mixture containing 25 mM NaCl, 100 μg/mL bovine serum albumin (BSA), and 2 mM dithiothreitol (DTT). A control reaction mixture was incubated under identical conditions with the exclusion of the enzyme. Reactions were terminated by addition of 5 × SDS-PAGE sample loading buffer (Beyotime, China) followed by heat treatment at 100°C for 10 min. Samples were analyzed by electrophoresis on a 12% SDS-polyacrylamide gel and stained with Coomassie blue dye. Reaction mixtures were boiled with sample buffer and then proteins were separated by SDS-PAGE.

### Assay of deubiquitination activity *in vivo*

HEK-293T cells cultured in 60-mm dishes were co-transfected with 1 μg of Flag-Ub, HA-K48-Ub, or HA-K63-Ub in addition to the appropriate amount of constructs containing USP25 or the corresponding mutants using Lipofectamine 2000. Where applicable, the empty pCAGGS-HA or pCAGGS-Myc vector was supplemented to keep the total amount of DNA transfected constant. After 30 h, cells were harvested by adding 200 μL 2 × lysis buffer A (LBA) (65 mM Tris-HCl (pH 6.8), 4% sodium dodecyl sulfate, 3% DL-dithiothreitol, and 40% glycerol) containing 20 mM N-ethylmaleimide (NEM) (Sigma-Aldrich) and 20 mM iodoacetamide (Sigma-Aldrich). Cell lysates were then analyzed for ubiquitin-conjugated proteins by Western blotting with anti-HA antibodies (1:1,000) (ABclonal Biotechnology) or anti-Flag antibodies (1:1,000) (Macgene, China). To confirm the expression levels of USP25 and the mutants, anti-HA antibodies were used to detect HA-tagged proteins, and anti-Myc antibodies (Beyotime, China) were used to detect Myc-tagged proteins. Beta-actin was detected with anti-beta-actin monoclonal antibodies (MAb) (Beyotime, China) to demonstrate equal protein sample loading.

### Co-immunoprecipitation and immunoblotting analysis

For transient transfection experiments, HEK-293T cells were transfected for 28 h. The transfected cells were lysed in 200 μL of lysis buffer (4% SDS, 3% DTT, 0.065 mM Tris-HCl, pH6.8, 30% glycerin) supplemented with protease inhibitors (PMSF). Lysates were boiled at 100°C for 10 min before being separated by SDS-PAGE and then electroblotted onto a polyvinylidene fluoride membrane (Bio-Rad), and analyzed by Western blotting with the indicated antibodies. Anti-USP25, anti-IRF3, anti-phosphor-IRF3, anti-NF-κB p65, anti-phosphor-NF-κB p65 and anti-ubiquitin antibodies were purchased from ABclonal Biotechnology. Horseradish peroxidase-conjugated anti-mouse or anti-rabbit IgG antibodies were obtained from Beyotime Institute of Biotechnology (Jiangsu, China).

For co-immunoprecipitation analysis, Cells were washed with phosphate-buffered saline and lysed for 20 min at 4°C in lysis buffer containing 50 mM Tris-HCl, pH 7.4, 150 mM NaCl, 1% NP40, 10% glycerin, 0.1% SDS, and 2 mM Na_2_EDTA. Lysates were then cleared by centrifugation, and proteins were immunoprecipitated with affinity antibodies and protein A+G agarose beads (Beyotime, China) at 4°C. Immunoprecipitates were washed three times with 1 mL of lysis buffer. The precipitates were analyzed by standard immunoblot procedures. 

### Statistical analysis

All experiments were performed at least three times with reproducible results. Data are presented as mean ± standard deviation (SD). Statistical analysis was performed using one-way analysis of variance (ANOVA) without interaction terms followed by Dunnett’s for multiple comparisons. A *P*-value of <0.05 was considered statistically significant.

## Results

### USP25 negatively regulates virus-induced activation of ISRE and the expression of ISGs

**Figure 1:**
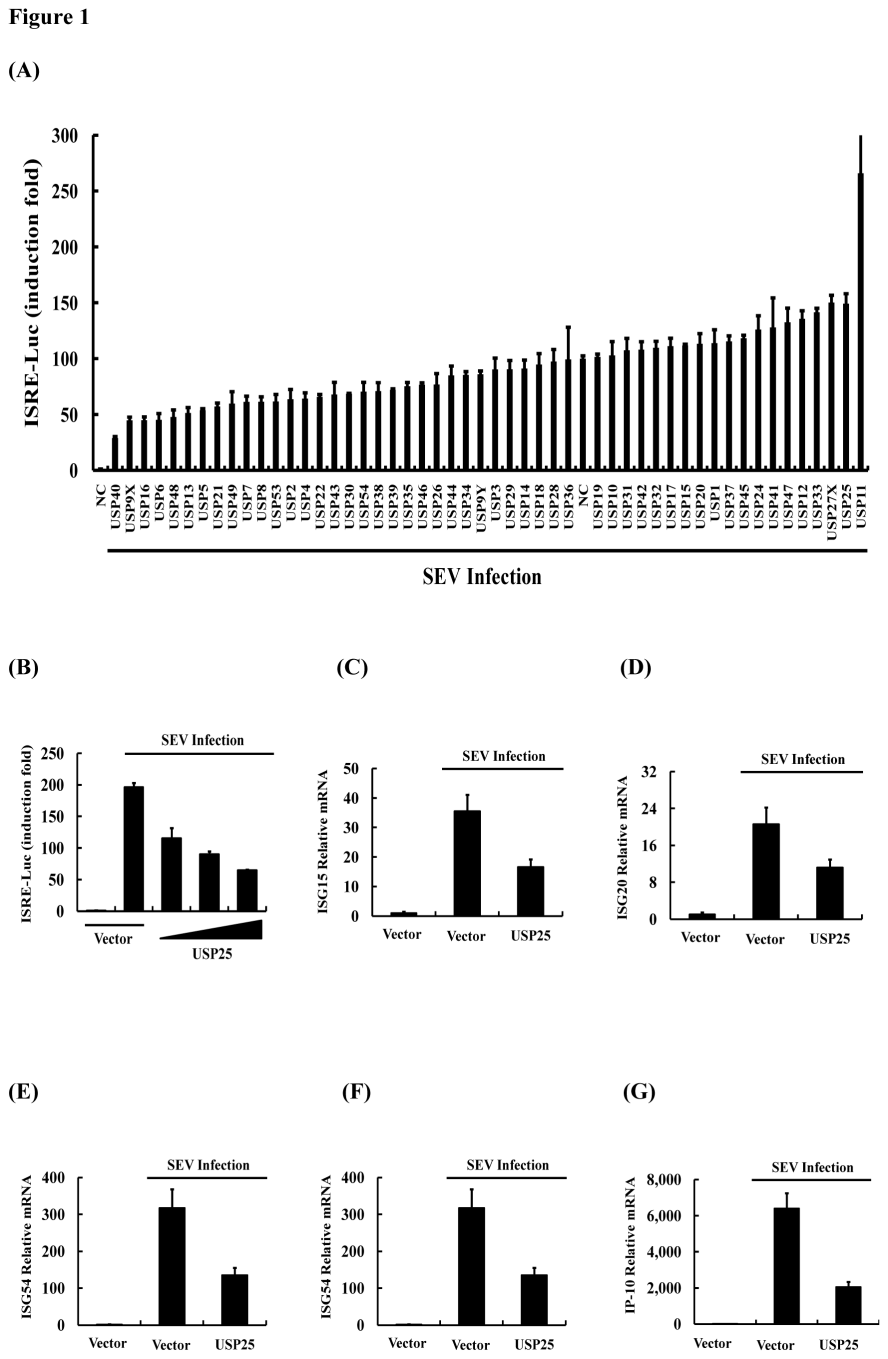
USP25 negatively regulates virus-induced activation of ISRE and the expression of ISGs. (A) A siRNA screen for USPs functions on SEV-induced activation of the ISRE promoter. HEK-293T cells were transfected with an ISRE luciferase reporter (0.1 μg) and control Renilla luciferase reporter (0.02 μg) vectors and NC (control siRNA) or specific siRNA pools (three siRNAs per gene) of members of USPs subclass of deubiquitinase for 24 h, and then infected or mock-infected with SEV for 16 h before luciferase assays were performed. (B) USP25 inhibited SEV-induced activation of ISRE. ISRE luciferase reporter (0.1 μg) and control Renilla luciferase reporter (0.02 μg) vectors were co-transfected into HEK-293T cells with either an empty vector (1 μg) or increasing amounts of USP25 (0.25, 0.5, or 1 μg) for 24 h. Cells were then either untreated or treated with SEV for 16 h before the relative luciferase activity was measured and normalized with the Renilla activity. Error bars indicate ± SD in three independent experiments. (C–G) USP25 significantly reduced the transcription of multiple ISGs. Empty vector (1 μg) or expression plasmid of USP25 (1 μg) were transfected into HEK-293T cells for 24 h and either untreated or treated with SEV for 16 h before real-time RT-PCR was performed.

As mentioned above, ISRE, as an IFN stimulation of promoter, plays a central role in the JAK-STAT signal transduction pathways, which leads to transcriptional induction of a wide range of downstream antiviral genes and the subsequent innate antiviral response. To identify potential USPs that might regulate the antiviral innate immune response, we screened a pool of 162 siRNAs targeting 54 human USPs genes for their abilities to regulate SEV-induced activation of the ISRE promoter in reporter assays in HEK-293T cells. HEK-293T cells were transfected with siRNA pools (three siRNAs per gene) as well as ISRE luciferase reporter plasmid. Twenty-four hours after transfection, cells were either uninfected or infected with SEV for 16 h before reporter assays were performed. As listed in Figure 1A, several siRNA pools potently potentiated SEV-induced activation of the ISRE promoter. Our further analysis focused on USP25, since a previous study demonstrated that USP11, which is the top-ranking candidate in this study, was constitutively associated with IκBα and attenuated IκBα degradation to negatively regulate TNFα-induced NF-κB activation [34].

To validate the effect of USP25 knockdown on the SEV-induced ISRE reporter activity, we further assessed whether overexpression of USP25 also affects ISRE reporter activity. HEK-293T cells were transfected with ISRE reporter plasmid and increasing amounts of HA-USP25 expression plasmids. The results suggest that overexpression of USP25 strongly inhibited SEV-induced activation of ISRE promoter in a dose-dependent manner (Figure 1B).

We next asked whether overexpression of USP25 affects the expression of ISGs, whose promoters contain the ISRE binding domain. To this end, HEK-293T cells were treated with SEV, and ISG expression was assessed after 16 h. We specifically measured the effect of overexpression of USP25 on mRNA levels of five ISGs (ISG15, ISG20, ISG54, ISG56 and IP-10). Real-time RT-PCR analysis suggested that the mRNA levels of ISG15 (Figure 1C), ISG20 (Figure 1D), ISG54 (Figure 1E), ISG56 (Figure 1F) and IP-10 (Figure 1G) were markedly reduced after stimulation in HEK-293T cells. These results suggest that USP25 indeed negatively regulates the antiviral innate immune response.

### USP25 Impacts On the Antiviral Innate Immune Response by Reduction of IFN-β Expression

**Figure 2:**
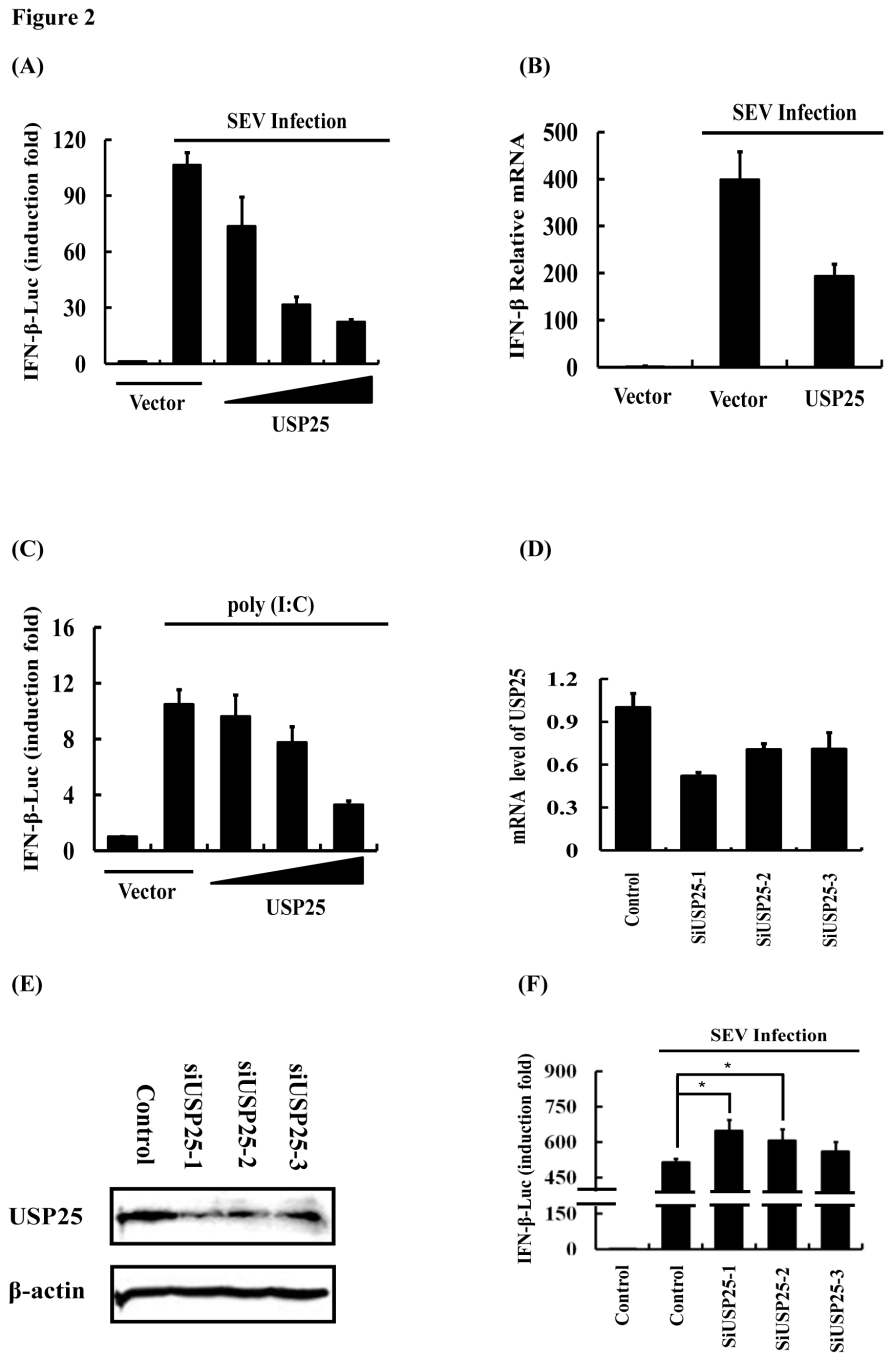
USP25 significantly inhibits virus-induced type I IFN signaling. (A) USP25 inhibited SEV-induced activation of the IFN-β promoter in a dose-dependent manner. IFN-β luciferase reporter (0.1 μg) and control Renilla luciferase reporter (0.02 μg) vectors were co-transfected into HEK-293T cells with an empty vector (1 μg) or increasing amounts of USP25 (0.25, 0.5, or 1 μg) for 24 h. Cells were then either untreated or treated with SEV for 16 h before the relative luciferase activity was measured and normalized with the Renilla activity. Error bars indicate ± SD in three independent experiments. (B) USP25 significantly inhibited the transcription of IFN-β. Empty vector (1 μg) or expression plasmid of USP25 (1 μg) were transfected into HEK-293T cells for 24 h and either untreated or treated with SEV for 16 h before real-time RT-PCR was performed. (C) USP25 inhibited poly (I:C)-induced activation of the IFN-β promoter in a dose-dependent manner. IFN-β luciferase reporter (0.1 μg) and control Renilla luciferase reporter (0.02 μg) vectors were co-transfected into HEK-293T cells with either an empty vector (1 μg) or increasing amounts of USP25 (0.25, 0.5, or 1 μg) for 24 h. Cells were then either untreated or treated with 1 μg of poly (I:C) for 16 h before the relative luciferase activity was measured and normalized with the Renilla activity. Error bars indicate ± SD in three independent experiments. (D, E) Effects of USP25 siRNA on endogenous USP25. HEK-293T cells were transfected with the indicated siRNA (20 nm each) for 24 h, and cell lysates were analyzed by quantitative reverse transcription (RT)-PCR (D) or immunoblots with antibodies against USP25 and beta-actin (E). (F) Effects of USP25 siRNA on SEV-induced activation of the IFN-β promoter. HEK-293T cells were transfected with an IFN-β luciferase reporter (0.1 μg) and control Renilla luciferase reporter (0.02 μg) vectors and the indicated siRNA plasmids (20 nm each) for 24 h and then infected with SEV or uninfected for 16 h before luciferase assays were performed. Error bars indicate ± SD in three independent experiments. *P < 0.05 compared with cells transfected with Control followed by SEV infection. Significant differences between groups were determined by one-way ANOVA followed by Dunnett’s multiple comparisons test.

It is well known that the IFN system constitutes the primary defense against viral infections. Therefore, we investigated the effect of overexpression of USP25 on virus-induced type I IFN signaling. We used an IFN-β-dependent luciferase reporter assay to assess its abilities to modulate SEV-induced IFN-β promoter in HEK-293T cells. The results showed that USP25 significantly reduced SEV-induced IFN-β luciferase reporter activity (Figure 2A). Consistent with these observations, quantitative reverse transcription (RT)-PCR showed that overexpression of USP25 inhibited SEV-induced expression of IFN-β (Figure 2B). In addition, poly (I:C)-induced production of IFN-β was significantly attenuated by the expression of USP25 (Figure 2C). We further investigated the effect of USP25 knockdown on virus-triggered IFN signaling. And we first tested the effect of individual siRNAs targeting USP25 by quantitative reverse transcription (RT)-PCR and immunoblotting. The results showed that expression of cell-endogenous USP25 were reduced both at the level of mRNA and protein (Figure 2D and E). Reporter assays then indicated that knockdown of USP25 markedly potentiated SEV-induced activation of the IFN-β promoter (Figure 2F). Collectively, these findings suggest that USP25 inhibits RIG-I/MDA5-dependent type I IFN signaling.

### USP25 inhibits SEV-induced type I IFN signaling by disrupting activation of IRF3 and NF-κB

**Figure 3:**
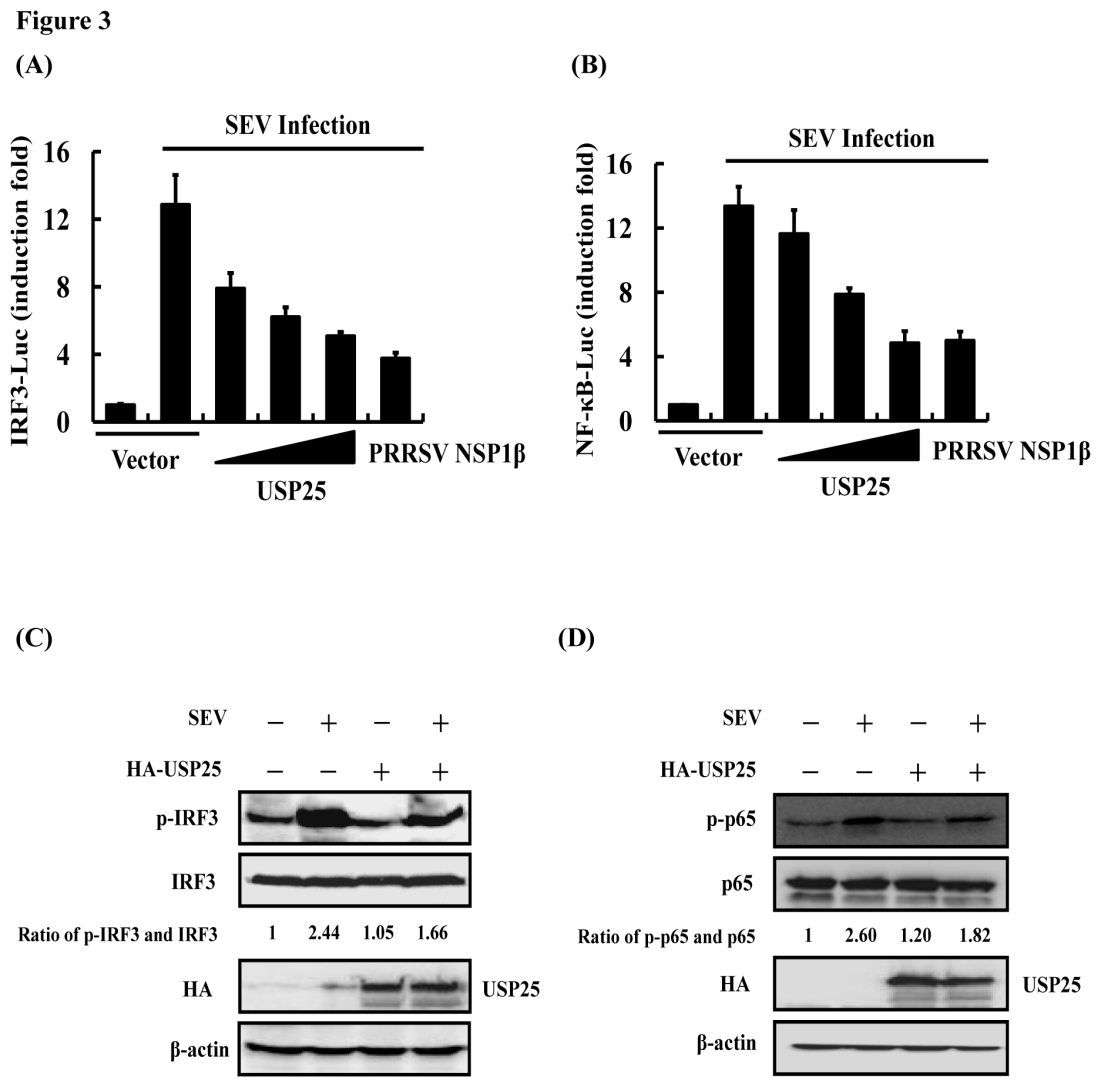
USP25 inhibits SEV-induced activation of IRF3 and NF-κB. (A) IRF3 luciferase reporter (0.1 μg) and control Renilla luciferase reporter (0.02 μg) vectors were co-transfected into HEK-293T cells with either an empty vector (1 μg) or increasing amounts of USP25 (0.25, 0.5, or 1 μg) or porcine reproductive and respiratory syndrome virus (PRRSV) NSP1β which was a positive control for 24 h. Cells were then either untreated or treated with SEV for 16 h before the relative luciferase activity was measured and normalized with the Renilla activity. Error bars indicate ± SD in three independent experiments. (B) Reporter assays were performed similarly as in A, except that different reporter plasmids were used. (C, D) USP25 inhibited SEV-induced IRF3 and p65 phosphorylation. HEK-293T cells were transfected with either an empty vector (3 μg) or expression plasmid of USP25 (3 μg) for 24 h. Cells were then either untreated or treated with SEV for 16 h and subsequently immunoblotted with the antibodies indicated. Quantification of the intensity of each band is listed under each band. Total protein of IRF3 and p65 was used as an internal control.

To better understand the effects of USP25 in SEV-induced type I IFN signaling, we assessed whether expression of USP25 disrupts SEV-induced activation of IRF3 and NF-κB, the two important transcriptional factors in type I IFN signaling. We observed that overexpression of USP25 significantly inhibited SEV-induced activation of IRF3 and NF-κB (Figure 3A and B). Previous study indicated that porcine reproductive and respiratory syndrome virus (PRRSV) NSP1β could inhibited both IFN regulatory factor 3 (IRF3)- and NF-κB-dependent gene induction by dsRNA and Sendai virus [35]. Therefore, we used NSP1β as a positive control in Figure 3A and B. We further examined whether USP25 affects virus-induced phosphorylation of IRF3 and p65. The results showed that overexpression of USP25 downregulated SEV-induced phosphorylation of IRF3 and p65 (Figure 3C and D). Taken together, our results suggest that USP25 negatively regulates IFN-β expression by inhibiting SEV-induced activation of IRF3 and NF-κB.

### USP25 processes K48-linked and K63-linked polyubiquitin *in vitro* and *in vivo*

**Figure 4:**
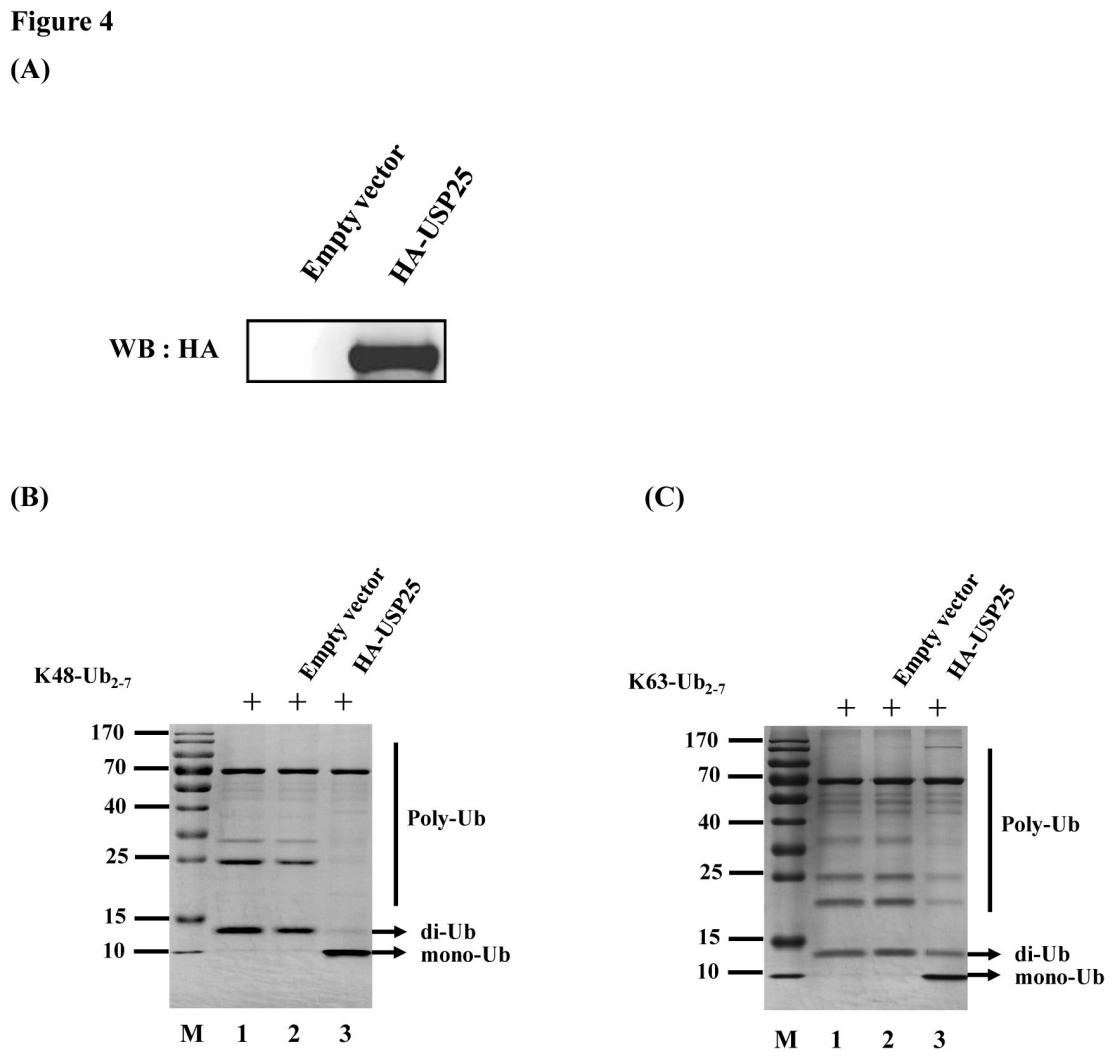
Processing of K48- and K63-linked polyubiquitin chains by USP25 *in* *vitro*. (A) Analysis for purified HA-tagged USP25 conjugated proteins. The protein was obtained from USP25-transfected or mock-transfected HEK-293T cells using a HA tagged Protein PURIFICATION KIT (MBL) and analyzed for HA-tagged USP25-conjugated proteins by western blotting (WB) with an anti-HA antibody. (B) *In* *vitro* K48-linked polyubiquitin deconjugation assay. K48-linked polyubiquitin was incubated with the protein obtained from mock-transfected (lane 2) or USP25-transfected (lane 3) HEK-293T cells at 37°C for 1 h before being analyzed by SDS-PAGE. Lane 1, uncleaved K48-linked polyubiquitin chain (K48-Ub_2–7_). M, molecular mass markers, including 170-, 130-, 100-, 70-, 55-, 40-, 35-, 25-, 15-, and 10-kDa bands. (C) *In* *vitro* K63-linked polyubiquitin deconjugation assay. The experiment was performed similarly as in B, except that the K63-linked polyubiquitin chain (K63-Ub_2–7_) was used.

USPs are cysteine proteases that vary greatly in size and structural complexity. Thus, it is unlikely that all predicted USPs are truly specific for Ub [23]. To identify if USP25 has DUB activity, a DNA construct expressing USP25 was transiently transfected into HEK-293T cells and the recombinant USP25 was purified from cell lysates using a HA tagged Protein PURIFICATION KIT (MBL) (Figure 4A). When incubated with K48- and K63-linked polyubiquitin chains *in vitro*, the purified USP25 was highly effective in cleaving both substrates into monomers (Figure 4B and C).

**Figure 5:**
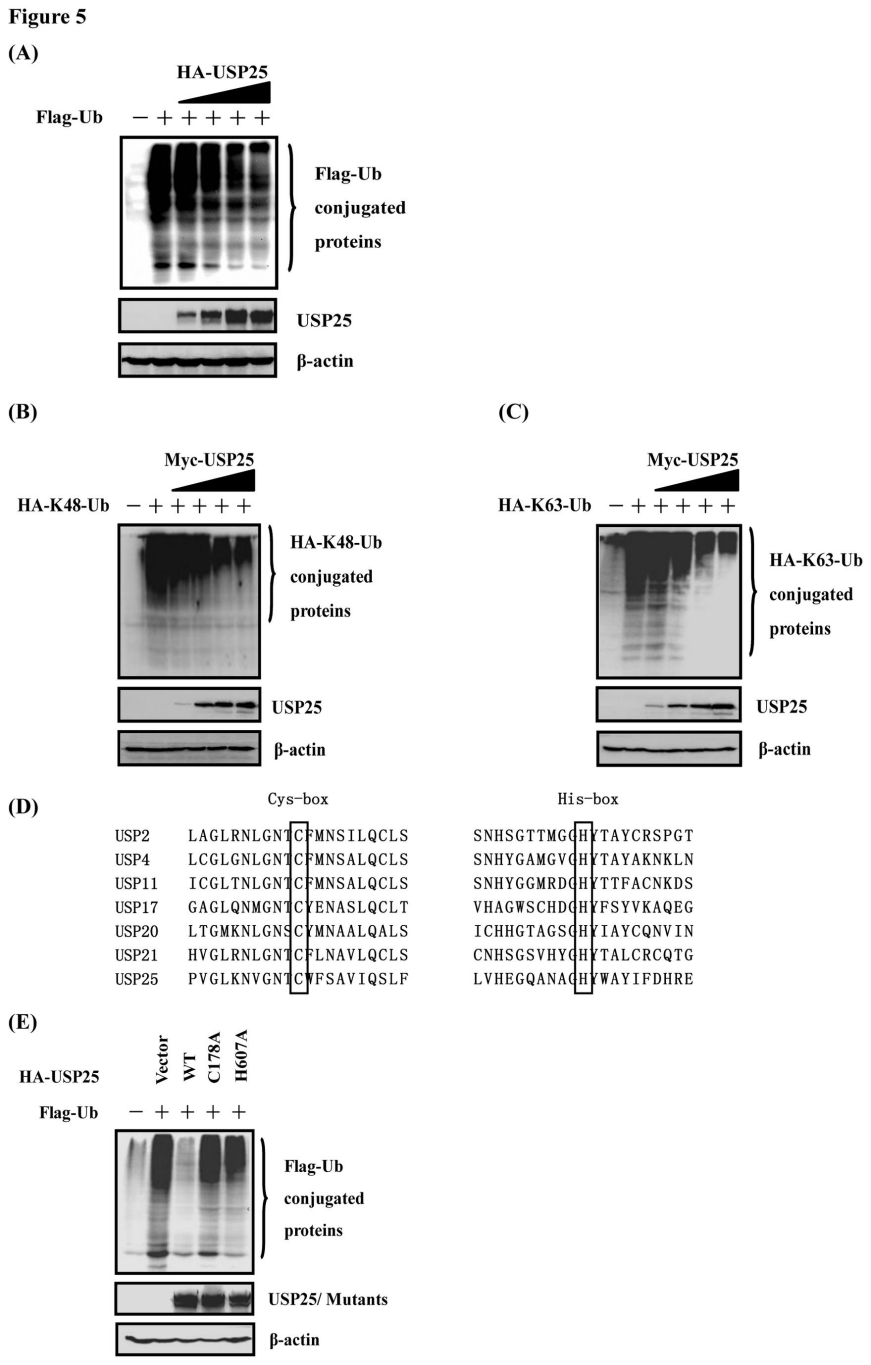
USP25 has a dose-dependent deubiquitinating activity *in* *vivo*. (A) HEK-293T cells grown in 60-mm dishes were transfected with Flag-tagged Ub expression plasmids (1 μg), along with increasing quantities (0.25, 0.5, 1, or 2 μg) of plasmid encoding USP25 using Lipofectamine 2000. Cell lysates were prepared at 30 h post-transfection and analyzed for Ub-conjugated proteins by Western blotting with an anti-Flag antibody. Western blotting with anti-HA antibodies shows expression of USP25, and Western blotting for beta-actin served as a protein loading control. (B, C) USP25 effectively cleaved both K48 and K63 Ub linkages *in* *vivo*. The experiment was performed similarly to that described for panel A except that HA-K48-Ub or HA-K63-Ub was used in lieu of Flag-Ub and different expression plasmids of USP25 were used. (D) Mapping of the putative sites are associated with the DUB activity of USP25. Black boxes indicate conserved residues tested in this study. The sequences were derived from GenBank entries with the following accession numbers: USP2, NM_004205; USP4, NM_003363; USP11, NM_004651; USP17, NM_201402 and XM_352721; USP20, NM_001110303; USP21, NM_001014443; and USP25, NM_013396. (E) Cysteine 178 (C178) and histidine 607 (H607) are deubiquitination active sites of USP25. Expression vectors encoding pcDNA3.1-Flag-Ub and control vectors or expression vectors encoding HA-USP25-WT, -C178A and -H607A were co-transfected into HEK-293T cells. USP25-WT and deletion mutant proteins in cell lysates were immunoprecipitated with anti-HA antibodies under denaturing conditions and immunoblotted with anti-Flag antibodies to detect the presence of Ub-conjugated proteins. Western blotting for beta-actin served as a protein loading control.

To further determine whether USP25 has DUB activity in a cell-based assay, HEK-293T cells were transfected with either an empty vector or increasing amounts of plasmid DNA encoding USP25 along with a Flag-tagged ubiquitin vector (Flag-Ub), and the effect of USP25 on all ubiquitinated cellular proteins was assessed via Western blotting with an anti-Flag antibody. As shown in Figure 5A, overexpression of USP25 resulted in a dose-dependent reduction in the levels of ubiquitinated cellular proteins compared with those levels observed in the control vector-transfected cells. To further identify which Ub linkage type is targeted by USP25 *in vivo*, HEK-293T cells were transfected with HA-K48-Ub or HA-K63-Ub in lieu of HA-Ub. These constructs allow solely for the formation of K48- and K63-linked polyubiquitin chains, respectively. As shown in Figure 5B and C, transfection with USP25 resulted in an obvious reduction in the extent of conjugation of K48- and K63-linked polyubiquitin. These observations, together with the results from the deubiquitination assays *in vitro*, clearly demonstrated that USP25 possesses DUB activity and affects both K48-linked and K63-linked ubiquitination.

### Identification of USP25 DUB activity sites

Based on the structures of their catalytic domains, the human DUBs have been classified into five subfamilies, most of which exhibit a high degree of homology mainly in two regions known as Cys and His boxes (C and H boxes, respectively) that surround the catalytic Cys and His residues [23,36]. We chose six USPs whose DUB activity had been reported [34,37–41]. Sequence alignment showed that Cys178 and His607 of USP25 are highly conserved among all seven ubiquitin-specific peptidase (USPs) (Figure 5D). Replacement of the putative catalytic cysteine residue (Cys178) and histidine residue (His 607) of USP25 by an alanine residue resulted in nearly total loss of its catalytic activity in contrast to USP25 wild-type (Figure 5E). This indicates that the enzymatic activity of USP25 is dependent on its catalytic site—cysteine residue (Cys178) and histidine residue (His607).

### Mutation of the catalytic residues results in significant loss of ability of USP25-mediated IFN inhibition

**Figure 6:**
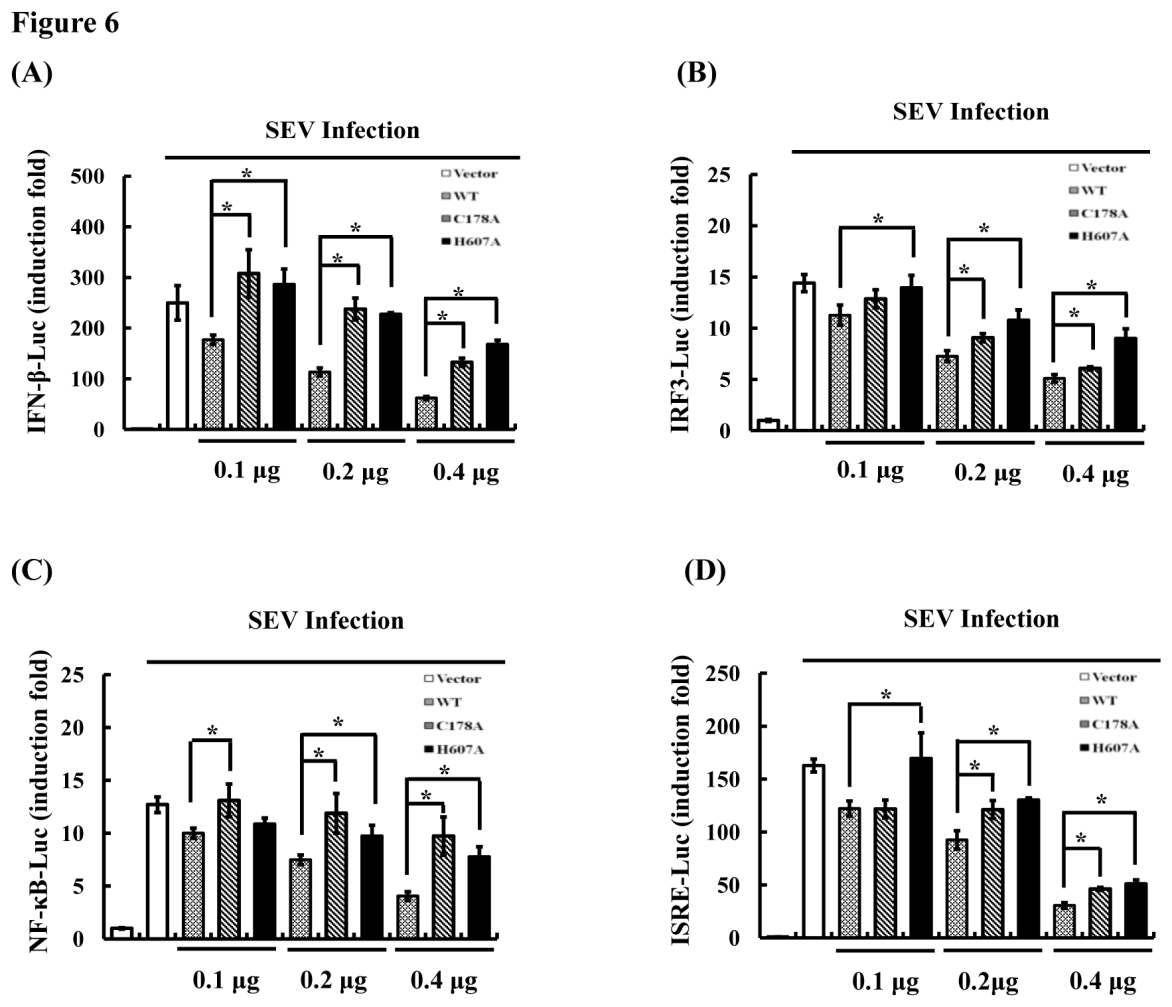
The deubiquitinating activity of USP25 is involved in virus-induced type I IFN signaling. (A) The deubiquitinating activity of USP25 was involved in the transcription of IFN-β. IFN-β luciferase reporter (0.1 μg) and control Renilla luciferase reporter (0.02 μg) vectors were co-transfected into HEK-293T cells with either an empty vector or increasing amounts of USP25-WT or deletion mutants for 24 h. Cells were then either untreated or treated with SEV for 16 h before the relative luciferase activity was measured and normalized with the Renilla activity. Error bars indicate ± SD in three independent experiments. *P < 0.05 for all pairwise comparisons by one-way ANOVA followed by Dunnett’s multiple comparisons test. (B–D) The deubiquitinating activity of USP25 was involved in the activation of IRF3 (B), NF-κB (C) and ISRE (D). Reporter assays were performed similarly as in A, except that different reporter plasmids were used. *P < 0.05 for all pairwise comparisons by one-way ANOVA followed by Dunnett’s multiple comparisons test.

To examine whether the inhibitory effects of USP25 on SEV-induced type I IFN signaling is due to its deubiquitinase activity, wild-type USP25 (USP25-WT) and its mutants (C178A and H607A) lacking DUB activity were co-transfected with the promoter luciferase reporter plasmid of IFN-β, IRF3, NF-κB and ISRE, and the luciferase activity was detected. The results showed that USP25-WT remarkably inhibited SEV-induced activation of IFN-β (Figure 6A), IRF3 (Figure 6B), NF-κB (Figure 6C) and ISRE (Figure 6D) in a dose-dependent manner. However, the catalytic mutants (C178A and H607A) devoid of DUB activity lost the ability of USP25 WT-mediated IFN inhibition to some degree, indicating that DUB activity is involved in USP25 inhibition of type I IFN induction.

### USP25 deubiquitinates RIG-I, TRAF2 and TRAF6

**Figure 7:**
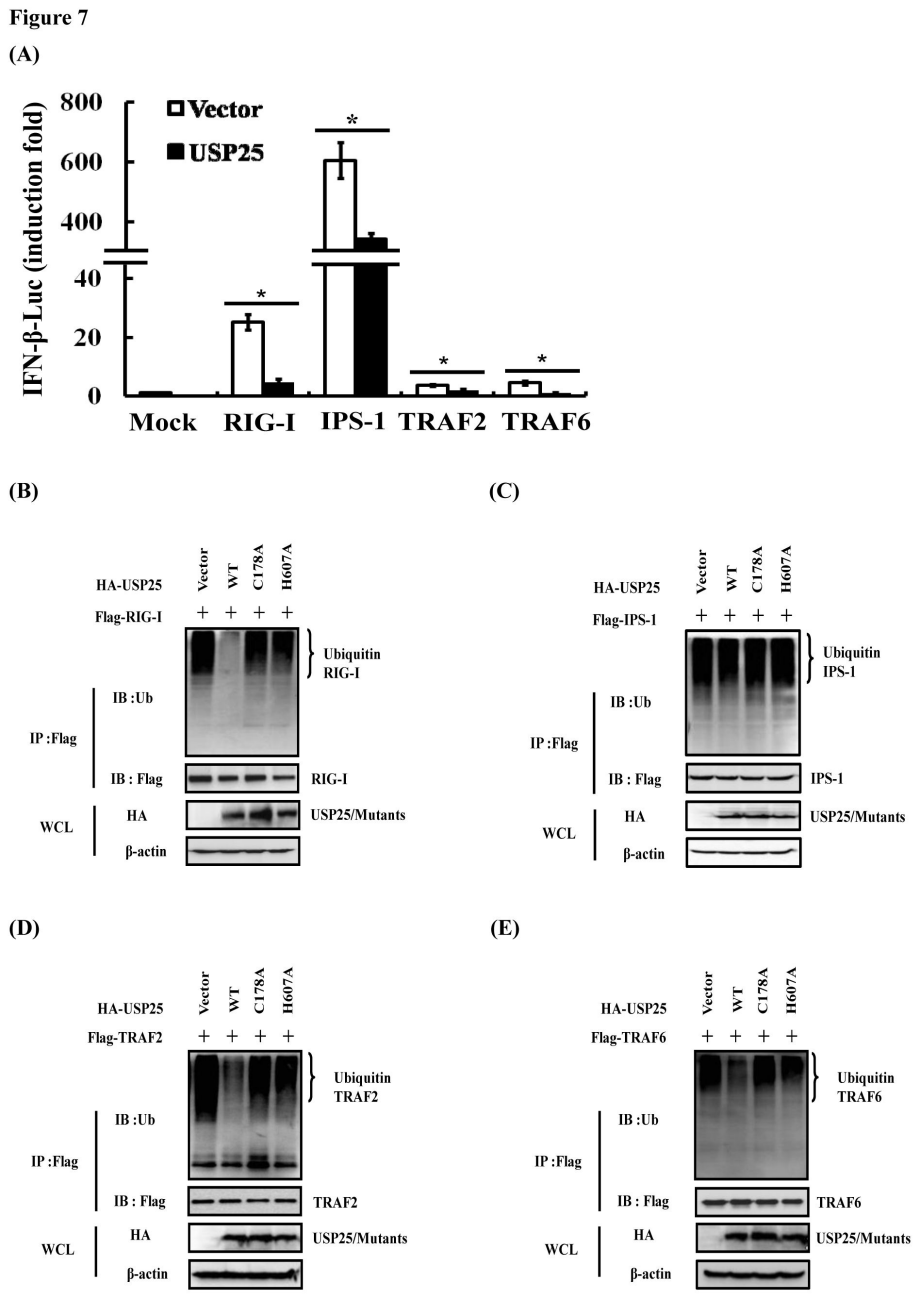
USP25 deubiquitinates RIG-I, TRAF2 and TRAF6. (A) USP25 inhibited RIG-I-, IPS-1-, TRAF2-, and TRAF6-mediated activation of the IFN-β promoter. HEK-293T cells were transfected with an IFN-β promoter reporter (0.1 μg) and either an empty vector or the indicated plasmids encoding the RIG-I, IPS-1, TRAF2, and TRAF6 expression vector (1 μg each) for 30 h before luciferase assays were performed. *p < 0.05 for all pairwise comparisons by one-way ANOVA followed by Dunnett’s multiple comparisons test. (B–E) HEK-293T cells were co-transfected with the indicated plasmids encoding the RIG-I (B), IPS-1 (C), TRAF2 (D), or TRAF6 (E) expression vector (4 μg) and HA-USP25WT/ HA-USP25C178A/ HA-USP25H607A (4 μg) using Lipofectamine 2000. The immunoprecipitates (IP) were analyzed by immunoblots (IB) with anti-ubiquitin (top panels) and anti-Flag (middle panels). The levels of the transfected USP25/mutants were detected by immunoblots with anti-HA (bottom panels). The input tagged proteins were verified with the indicated antibodies.

To further determine the levels at which USP25 negatively regulates type I IFN signaling, HEK-293T cells were transfected with DNA constructs encoding RIG-I, IPS-1, TRAF2, or TRAF6, together with IFN-β-Luc. As shown in Figure 7A, overexpression of RIG-I, IPS-1, TRAF2, or TRAF6 significantly activated IFN-β promoter compared with cells transfected with an empty vector control. However, such effects were all substantially reduced in the presence of USP25.

Since the DUB activity of USP25 was involved in virus-induced type I IFN signaling, we further investigated whether the IFN antagonist function of USP25 is associated with the deubiquitination of RIG-I, IPS-1, TRAF2, and TRAF6, which are essential signaling components in the type I IFN pathway activated by virus infection. We observed that overexpression of USP25 significantly inhibited ubiquitination of RIG-I (Figure 7B), TRAF2 (Figure 7D), and TRAF6 (Figure 7E). In contrast, the USP25 mutants (C178A and H607A) lacking DUB activity had no such effects. However, overexpression of USP25 did not block ubiquitination of IPS-1 (Figure 7C). Taken together, our results confirmed that USP25 acts as a deubiquitinase of RIG-I, TRAF2 and TRAF6 to inhibit virus-induced type I IFN signaling.

## Discussion

The production of type I IFNs and induction of interferon-inducible genes after virus infection are central to antiviral innate immune responses. Ubiquitination and deubiquitination have emerged as critical post-translational regulatory mechanisms for activation or attenuation of the virus-triggered IFN response pathway. Previous studies have demonstrated a critical role for the members of DUBs in antiviral innate immune responses. For example, several deubiquitinating enzymes, including A20, Cezanne, CYLD, USP15, and USP31, have been suggested to be involved in the downregulation of TNF-α induced NF-κB activation [27,42–45]. In recent years, the USPs subclass of DUBs has been deemed to represent the bulk of the deubiquitinating enzymes encoded in the human genome, and their distribution and functional diversity in eukaryotic tissues and organs have received extensive attention. However, the roles of USPs in the regulation of type I IFN production have not been studied in great detail. In this study, we identified USP25 as a major deubiquitinase of RIG-I, TRAF2, and TRAF6 in SEV-induced type I IFN induction.

Of the DUBs family, USPs have raised special interest because of the multiple family members described in different eukaryotic organisms. It is thought that not all USPs that contain well-conserved motifs have DUB activity. Recently, Quesada et al. reported that USP40, USP47, and USP48 show no activity, despite possessing all the catalytic residues proposed as being important for the catalytic activity of these enzymes [46]. However, DUB activity had been demonstrated for many USPs. For example, Junichiro et al. demonstrated that USP21 constitutively deubiquitinates RIP1 *in vitro* and *in vivo* [40]; and a previous study has reported that USP20 deubiquitinates TRAF6 and Tax *in vivo* [40]. Here, we found that USP25 contains conserved Cys178 and His607 residues, which are important for the catalytic activity of USPs, suggesting that USP25 may have deubiquitinating activity. In subsequent experiments, we observed that USP25 did indeed appear to have DUB activity both *in vitro* and *in vivo*. We also revealed that USP25 could act on both K48- and K63-linked Ub polymers. In addition, we also found that Cys178 and His607 residues are responsible for the DUB activity of USP25.

By using a siRNA screen, we found that knockdowns of several USPs potently potentiated SEV-induced activation of the ISRE promoter, some of which had been reported as being associated with the NF-κB signaling pathway. For example, USP11 negatively regulates TNFα-induced NF-κB activation associated with IκBα and attenuates IκBα degradation [34]; USP20 deubiquitinates TRAF6 and suppresses interleukin 1β (IL-1β)- and Tax-induced NF-κB activation [40]; Katrin et al. showed that USP15 regulates IκBα/NF-κB by deubiquitinylation IκBα[44]; and USP31 inhibits TNFα, CD40, TRAF2, TRAF6 and IKKβ-mediated NF-κB activation [45]. 

It is well known that the IFN-β promoter contains conserved enhancer elements recognized by NF-κB that lead to induction of IFN-β, which then activates ISRE. Thus, these USPs (USP11, USP20, USP15, and USP31) are very likely to inhibit virus-induced ISRE reporter activity. Consistent with this supposition, we found that knockdowns of these USPs strengthened SEV-induced ISRE reporter activity. In this study we focus on USP25, whose knockdown significantly potentiated SEV-induced activation of the ISRE promoter in the siRNA screen. However, this screening serves only as an initial step to identify the genes. We further found that overexpression of USP25 efficiently reduced SEV-induced IFN-β induction.

Ubiquitination and deubiquitination are critical players in modulating the antiviral innate immune response. Several ubiquitin ligase enzymes have been found to regulate these processes [47,48]. For example, ubiquitination of RIG-I by the E3 ubiquitin ligase TRIM25, which contains a RING finger domain, is necessary and sufficient to activate IPS-1, which triggers the downstream signaling cascade to produce type I IFN [49]. Virus-triggered ubiquitination of TRAF2/6 by cIAP1/2 is essential for induction of IFN-β and the cellular antiviral response [50,51]. However, certain cellular USPs are known to modulate the antiviral innate immune response. For example, USP17 and USP20 were found to target RIG-I and TRAF6 respectively, thereby functioning as novel regulators of antiviral innate immune responses [39,40]. In the present study, we are the first to expound that USP25 inhibited RIG-I- , IPS-1-, TRAF2-, and TRAF6-mediated activation of the IFN-β promoter. In addition, wild-type USP25 significantly inhibits ubiquitination of RIG-I, TRAF2, and TRAF6, which is essential for activation of type I IFN signaling. However, both catalytically inactive mutants that are defective for DUB activity lost the capability of reducing ubiquitinated RIG-I, TRAF2, and TRAF6. In addition, the catalytic USP25 mutants (C178A and H607A) devoid of DUB activity significantly lost the ability of USP25 WT-mediated IFN inhibition. Taken together, these results indicate that the DUB activity of USP25 is involved in the inhibition of type I IFN induction.

However, inhibition of DUB activity by mutagenesis means did not completely abrogate the ability of USP25 to block viral activation of the type I IFN signaling pathway. We hypothesize that mutation of the catalytic residues does not abolish the ability to combine with ubiquitin of target proteins; and that it is this kind of combination that may reduce the ability of proteins to transmit signals without cleaving the ubiquitin chains. Indeed, co-immunoprecipitation investigation showed that USP25 interacted with RIG-I and TRAF6 (see Figure S1). Therefore, we hypothesize that USP25 may affect RIG-I-mediated signal transmission by interacting with RIG-I and TRAF6 to reduce type I interferon induction. Further investigation will be required to identify USP25 mutants that disrupt the interaction of USP25 and RIG-I/TRAF6 but do not affect DUB activity; and to test whether these domains of USP25 interacting with RIG-I and TRAF6 also contribute to the inhibition of IFN induction by USP25.

In recent years, USP25 has been extensively studied and some novel functions have been revealed. Previous studies have shown that the DUB activity of USP25m, a muscle isoform of USP25, did not strictly depend on the ubiquitin-binding domains (UBDs), but required a coiled-coil stretch between amino acids 679 to 769 [52]. Future investigation will be required to test whether these domains of USP25 are also involved in USP25-mediated IFN inhibition. In addition, USP25 interacts with the ubiquitin ligase HRD1 and rescues several endoplasmic reticulum-associated degradation (ERAD) substrates from degradation by the proteasome [53]. Also, USP25 can be a functional deubiquitinase of TRAF5 and TRAF6 in the regulation of IL-17-mediated signaling and inflammation [54]. Recently Zhong et al. reported that USP25 was a critical modulator of TLR4-mediated, but not TLR3-mediated, signaling. They mainly focus on the regulation of USP25 deubiquitination in TLR4 signaling in terms of the production of proinflammatory cytokines and type I IFNs [55]. However, our studies explain that USP25 negatively modulates RIG-I/MDA5-dependent type I IFN signaling. These have showed that USP25 has a wide range of biological activity and differentially regulates innate response.

 In our study, we observed USP25 to act as a DUB that cleaved ubiquitin chains from RIG-I, TRAF2, and TRAF6, thereby inhibiting SEV-induced type I IFN signaling. In conclusion, our studies suggest that USP25 serves as another level of critical regulatory control to maintain a delicate balance in virus-induced type I IFN signaling.

## Supporting Information

Figure S1 **USP25 interacts with RIG-I and TRAF6.** (A, B) HEK-293T cells grown in 100-mm dishes were co-transfected with the indicated plasmids encoding the RIG-I (A) or TRAF6 (B) expression vector (4 μg) and HA-USP25WT/ HA-USP25C178A/ HA-USP25H607A (4 μg) using Lipofectamine 2000. Immunoprecipitation (IP) was analyzed by immunoblots (IB) with anti-HA (top panels) and anti-Flag (middle panels). The input tagged proteins were verified with the indicated antibodies.
(TIF)

Table S1 **Sequence for siRNAs of human USP25.**
(DOC)

Table S2 **Primers for effective genes of innate immunity used in real-time RT-PCR.**
(DOC)

## References

[1] Kawai T, Akira S (2006) Innate immune recognition of viral infection. Nat Immunol 7: 131-137. doi:10.1038/ni1303. PubMed: 16424890. 16424890

[2] Darnell JE Jr., Kerr IM, Stark GR (1994) Jak-STAT pathways and transcriptional activation in response to IFNs and other extracellular signaling proteins. Science 264: 1415-1421. doi:10.1126/science.8197455. PubMed: 8197455. 8197455

[3] Hubackova S, Novakova Z, Krejcikova K, Kosar M, Dobrovolna J et al. (2010) Regulation of the PML tumor suppressor in drug-induced senescence of human normal and cancer cells by JAK/STAT-mediated signaling. Cell Cycle 9: 3085-3099. doi:10.4161/cc.9.15.12521. PubMed: 20699642. 20699642

[4] Kato H, Takeuchi O, Sato S, Yoneyama M, Yamamoto M et al. (2006) Differential roles of MDA5 and RIG-I helicases in the recognition of RNA viruses. Nature 441: 101-105. doi:10.1038/nature04734. PubMed: 16625202. 16625202

[5] Pichlmair A, Schulz O, Tan CP, Näslund TI, Liljeström P et al. (2006) RIG-I-mediated antiviral responses to single-stranded RNA bearing 5'-phosphates. Science 314: 997-1001. doi:10.1126/science.1132998. PubMed: 17038589. 17038589

[6] Ye J, Maniatis T (2011) Negative regulation of interferon-beta gene expression during acute and persistent virus infections. PLOS ONE 6: e20681. doi:10.1371/journal.pone.0020681. PubMed: 21677781. 21677781 PMC3108996

[7] Loo YM, Fornek J, Crochet N, Bajwa G, Perwitasari O et al. (2008) Distinct RIG-I and MDA5 signaling by RNA viruses in innate immunity. J Virol 82: 335-345. doi:10.1128/JVI.01080-07. PubMed: 17942531. 17942531 PMC2224404

[8] Maelfait J, Beyaert R (2012) Emerging role of ubiquitination in antiviral RIG-I signaling. Microbiol Mol Biol Rev 76: 33-45. doi:10.1128/MMBR.05012-11. PubMed: 22390971. 22390971 PMC3294425

[9] Seth RB, Sun L, Ea CK, Chen ZJ (2005) Identification and characterization of MAVS, a mitochondrial antiviral signaling protein that activates NF-kappaB and IRF 3. Cell 122: 669-682. doi:10.1016/j.cell.2005.08.012. PubMed: 16125763. 16125763

[10] Xu LG, Wang YY, Han KJ, Li LY, Zhai Z et al. (2005) VISA is an adapter protein required for virus-triggered IFN-beta signaling. Mol Cell 19: 727-740. doi:10.1016/j.molcel.2005.08.014. PubMed: 16153868. 16153868

[11] Kawai T, Takahashi K, Sato S, Coban C, Kumar H et al. (2005) IPS-1, an adaptor triggering RIG-I- and Mda5-mediated type I interferon induction. Nat Immunol 6: 981-988. doi:10.1038/ni1243. PubMed: 16127453. 16127453

[12] Meylan E, Curran J, Hofmann K, Moradpour D, Binder M et al. (2005) Cardif is an adaptor protein in the RIG-I antiviral pathway and is targeted by hepatitis C virus. Nature 437: 1167-1172. doi:10.1038/nature04193. PubMed: 16177806. 16177806

[13] Kato H, Takahasi K, Fujita T (2011) RIG-I-like receptors: cytoplasmic sensors for non-self RNA. Immunol Rev 243: 91-98. doi:10.1111/j.1600-065X.2011.01052.x. PubMed: 21884169. 21884169

[14] Yoneyama M, Fujita T (2007) RIG-I family RNA helicases: cytoplasmic sensor for antiviral innate immunity. Cytokine Growth Factor Rev 18: 545-551. doi:10.1016/j.cytogfr.2007.06.023. PubMed: 17683970. 17683970

[15] Weissman AM (2001) Themes and variations on ubiquitylation. Nat Rev Mol Cell Biol 2: 169-178. doi:10.1038/35056563. PubMed: 11265246. 11265246

[16] Reyes-Turcu FE, Ventii KH, Wilkinson KD (2009) Regulation and cellular roles of ubiquitin-specific deubiquitinating enzymes. Annu Rev Biochem 78: 363-397. doi:10.1146/annurev.biochem.78.082307.091526. PubMed: 19489724. 19489724 PMC2734102

[17] Wilkinson KD (2009) DUBs at a glance. J Cell Sci 122: 2325-2329. doi:10.1242/jcs.041046. PubMed: 19571111. 19571111 PMC2704873

[18] Song L, Rape M (2008) Reverse the curse--the role of deubiquitination in cell cycle control. Curr Opin Cell Biol 20: 156-163. doi:10.1016/j.ceb.2008.01.012. PubMed: 18346885. 18346885 PMC2387050

[19] Schmidt M, Hanna J, Elsasser S, Finley D (2005) Proteasome-associated proteins: regulation of a proteolytic machine. Biol Chem 386: 725-737. PubMed: 16201867. 16201867 10.1515/BC.2005.085

[20] Kayagaki N, Phung Q, Chan S, Chaudhari R, Quan C et al. (2007) DUBA: a deubiquitinase that regulates type I interferon production. Science 318: 1628-1632. doi:10.1126/science.1145918. PubMed: 17991829. 17991829

[21] Adhikari A, Xu M, Chen ZJ (2007) Ubiquitin-mediated activation of TAK1 and IKK. Oncogene 26: 3214-3226. doi:10.1038/sj.onc.1210413. PubMed: 17496917. 17496917

[22] Pickart CM (2001) Mechanisms underlying ubiquitination. Annu Rev Biochem 70: 503-533. doi:10.1146/annurev.biochem.70.1.503. PubMed: 11395416. 11395416

[23] Nijman SM, Luna-Vargas MP, Velds A, Brummelkamp TR, Dirac AM et al. (2005) A genomic and functional inventory of deubiquitinating enzymes. Cell 123: 773-786. doi:10.1016/j.cell.2005.11.007. PubMed: 16325574. 16325574

[24] McCloskey RJ, Kemphues KJ (2012) Deubiquitylation machinery is required for embryonic polarity in Caenorhabditis elegans. PLOS Genet 8: e1003092 PubMed: 23209443. 23209443 10.1371/journal.pgen.1003092 PMC3510043

[25] Bhoj VG, Chen ZJ (2009) Ubiquitylation in innate and adaptive immunity. Nature 458: 430-437. doi:10.1038/nature07959. PubMed: 19325622. 19325622

[26] Friedman CS, O'Donnell MA, Legarda-Addison D, Ng A, Cárdenas WB et al. (2008) The tumour suppressor CYLD is a negative regulator of RIG-I-mediated antiviral response. EMBO Rep 9: 930-936. doi:10.1038/embor.2008.136. PubMed: 18636086. 18636086 PMC2529351

[27] Wertz IE, O'Rourke KM, Zhou H, Eby M, Aravind L et al. (2004) De-ubiquitination and ubiquitin ligase domains of A20 downregulate NF-kappaB signalling. Nature 430: 694-699. doi:10.1038/nature02794. PubMed: 15258597. 15258597

[28] Ehrhardt C, Kardinal C, Wurzer WJ, Wolff T, von Eichel-Streiber C et al. (2004) Rac1 and PAK1 are upstream of IKK-epsilon and TBK-1 in the viral activation of interferon regulatory factor-3. FEBS Lett 567: 230-238. doi:10.1016/j.febslet.2004.04.069. PubMed: 15178328. 15178328

[29] Nishikawa H, Ooka S, Sato K, Arima K, Okamoto J et al. (2004) Mass spectrometric and mutational analyses reveal Lys-6-linked polyubiquitin chains catalyzed by BRCA1-BARD1 ubiquitin ligase. J Biol Chem 279: 3916-3924. PubMed: 14638690. 14638690 10.1074/jbc.M308540200

[30] Clementz MA, Chen Z, Banach BS, Wang Y, Sun L et al. (2010) Deubiquitinating and interferon antagonism activities of coronavirus papain-like proteases. J Virol 84: 4619-4629. doi:10.1128/JVI.02406-09. PubMed: 20181693. 20181693 PMC2863753

[31] Luo R, Xiao S, Jiang Y, Jin H, Wang D et al. (2008) Porcine reproductive and respiratory syndrome virus (PRRSV) suppresses interferon-beta production by interfering with the RIG-I signaling pathway. Mol Immunol 45: 2839-2846. doi:10.1016/j.molimm.2008.01.028. PubMed: 18336912. 18336912 PMC7112510

[32] Yoneyama M, Suhara W, Fukuhara Y, Fukuda M, Nishida E et al. (1998) Direct triggering of the type I interferon system by virus infection: activation of a transcription factor complex containing IRF-3 and CBP/p300. EMBO J 17: 1087-1095. doi:10.1093/emboj/17.4.1087. PubMed: 9463386. 9463386 PMC1170457

[33] Niwa H, Yamamura K, Miyazaki J (1991) Efficient selection for high-expression transfectants with a novel eukaryotic vector. Gene 108: 193-199. doi:10.1016/0378-1119(91)90434-D. PubMed: 1660837. 1660837

[34] Sun W, Tan X, Shi Y, Xu G, Mao R et al. (2010) USP11 negatively regulates TNFalpha-induced NF-kappaB activation by targeting on IkappaBalpha. Cell Signal 22: 386-394. doi:10.1016/j.cellsig.2009.10.008. PubMed: 19874889. 19874889 PMC2794974

[35] Beura LK, Sarkar SN, Kwon B, Subramaniam S, Jones C et al. (2010) Porcine reproductive and respiratory syndrome virus nonstructural protein 1beta modulates host innate immune response by antagonizing IRF3 activation. J Virol 84: 1574-1584. doi:10.1128/JVI.01326-09. PubMed: 19923190. 19923190 PMC2812326

[36] Wilkinson KD, Tashayev VL, O'Connor LB, Larsen CN, Kasperek E et al. (1995) Metabolism of the polyubiquitin degradation signal: structure, mechanism, and role of isopeptidase T. Biochemistry 34: 14535-14546. doi:10.1021/bi00044a032. PubMed: 7578059. 7578059

[37] Metzig M, Nickles D, Falschlehner C, Lehmann-Koch J, Straub BK et al. (2011) An RNAi screen identifies USP2 as a factor required for TNF-alpha-induced NF-kappaB signaling. Int J Cancer 29: 607-618. 10.1002/ijc.26124 21480224

[38] Fan YH, Yu Y, Mao RF, Tan XJ, Xu GF et al. (2011) USP4 targets TAK1 to downregulate TNFalpha-induced NF-kappaB activation. Cell Death Differ 18: 1547-1560. doi:10.1038/cdd.2011.11. PubMed: 21331078. 21331078 PMC3136563

[39] Chen R, Zhang L, Zhong B, Tan B, Liu Y et al. (2010) The ubiquitin-specific protease 17 is involved in virus-triggered type I IFN signaling. Cell Res 20: 802-811. doi:10.1038/cr.2010.41. PubMed: 20368735. 20368735

[40] Yasunaga J, Lin FC, Lu X, Jeang KT (2011) Ubiquitin-specific peptidase 20 targets TRAF6 and human T cell leukemia virus type 1 tax to negatively regulate NF-kappaB signaling. J Virol 85: 6212-6219. doi:10.1128/JVI.00079-11. PubMed: 21525354. 21525354 PMC3126525

[41] Xu G, Tan X, Wang H, Sun W, Shi Y et al. (2010) Ubiquitin-specific peptidase 21 inhibits tumor necrosis factor alpha-induced nuclear factor kappaB activation via binding to and deubiquitinating receptor-interacting protein 1. J Biol Chem 285: 969-978. doi:10.1074/jbc.M109.042689. PubMed: 19910467. 19910467 PMC2801298

[42] Enesa K, Zakkar M, Chaudhury H, Luong le A, Rawlinson L et al. (2008) NF-kappaB suppression by the deubiquitinating enzyme Cezanne: a novel negative feedback loop in pro-inflammatory signaling. J Biol Chem 283: 7036-7045. doi:10.1074/jbc.M708690200. PubMed: 18178551. 18178551

[43] Brummelkamp TR, Nijman SM, Dirac AM, Bernards R (2003) Loss of the cylindromatosis tumour suppressor inhibits apoptosis by activating NF-kappaB. Nature 424: 797-801. doi:10.1038/nature01811. PubMed: 12917690. 12917690

[44] Schweitzer K, Bozko PM, Dubiel W, Naumann M (2007) CSN controls NF-kappaB by deubiquitinylation of IkappaBalpha. EMBO J 26: 1532-1541. doi:10.1038/sj.emboj.7601600. PubMed: 17318178. 17318178 PMC1829370

[45] Tzimas C, Michailidou G, Arsenakis M, Kieff E, Mosialos G et al. (2006) Human ubiquitin specific protease 31 is a deubiquitinating enzyme implicated in activation of nuclear factor-kappaB. Cell Signal 18: 83-92. doi:10.1016/j.cellsig.2005.03.017. PubMed: 16214042. 16214042

[46] Quesada V, Díaz-Perales A, Gutiérrez-Fernández A, Garabaya C, Cal S et al. (2004) Cloning and enzymatic analysis of 22 novel human ubiquitin-specific proteases. Biochem Biophys Res Commun 314: 54-62. doi:10.1016/j.bbrc.2003.12.050. PubMed: 14715245. 14715245

[47] Isaacson MK, Ploegh HL (2009) Ubiquitination, ubiquitin-like modifiers, and deubiquitination in viral infection. Cell Host Microbe 5: 559-570. doi:10.1016/j.chom.2009.05.012. PubMed: 19527883. 19527883 PMC7103382

[48] Sun SC (2008) Deubiquitylation and regulation of the immune response. Nat Rev Immunol 8: 501-511. doi:10.1038/nri2337. PubMed: 18535581. 18535581 PMC5763493

[49] Gack MU, Shin YC, Joo CH, Urano T, Liang C et al. (2007) TRIM25 RING-finger E3 ubiquitin ligase is essential for RIG-I-mediated antiviral activity. Nature 446: 916-920. doi:10.1038/nature05732. PubMed: 17392790. 17392790

[50] Mao AP, Li S, Zhong B, Li Y, Yan J et al. (2010) Virus-triggered ubiquitination of TRAF3/6 by cIAP1/2 is essential for induction of interferon-beta (IFN-beta) and cellular antiviral response. J Biol Chem 285: 9470-9476. doi:10.1074/jbc.M109.071043. PubMed: 20097753. 20097753 PMC2843197

[51] Mace PD, Smits C, Vaux DL, Silke J, Day CL (2010) Asymmetric recruitment of cIAPs by TRAF2. J Mol Biol 400: 8-15. doi:10.1016/j.jmb.2010.04.055. PubMed: 20447407. 20447407

[52] Denuc A, Bosch-Comas A, Gonzàlez-Duarte R, Marfany G (2009) The UBA-UIM domains of the USP25 regulate the enzyme ubiquitination state and modulate substrate recognition. PLOS ONE 4: e5571. doi:10.1371/journal.pone.0005571. PubMed: 19440361. 19440361 PMC2679190

[53] Blount JR, Burr AA, Denuc A, Marfany G, Todi SV (2012) Ubiquitin-specific protease 25 functions in Endoplasmic Reticulum-associated degradation. PLOS ONE 7: e36542. doi:10.1371/journal.pone.0036542. PubMed: 22590560. 22590560 PMC3348923

[54] Zhong B, Liu X, Wang X, Chang SH, Wang A et al. (2012) Negative regulation of IL-17-mediated signaling and inflammation by the ubiquitin-specific protease USP25. Nat Immunol 13: 1110-1117. doi:10.1038/ni.2427. PubMed: 23042150. 23042150 PMC3477275

[55] Zhong B, Liu X, Wang X, Li H, Darnay BG et al. (2013) Ubiquitin-specific protease 25 regulates TLR4-dependent innate immune responses through deubiquitination of the adaptor protein TRAF3. Sci Signal 6: ra35–: ra35 PubMed: 23674823. 10.1126/scisignal.2003708 PMC5763475 23674823

