# Supplementary figures and images for: Ubiquitin-Specific Proteases 25 Negatively Regulates Virus-Induced Type I Interferon Signaling
Source: PLoS One. 2013 Nov 18;8(11):e80976. doi: 10.1371/journal.pone.0080976 (PMC3832446; doi:10.1371/journal.pone.0080976)

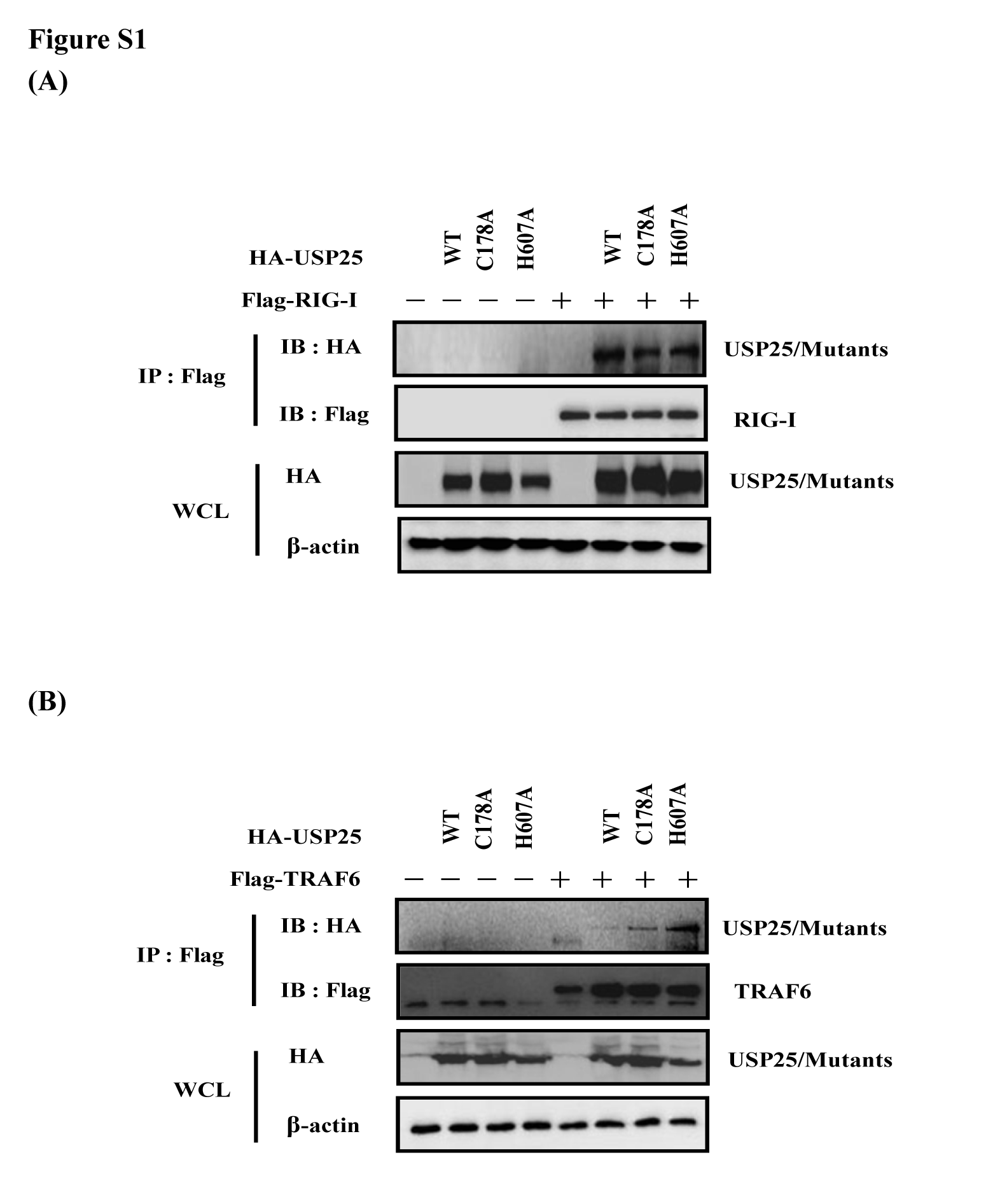

Supplement: Figure S1 — USP25 interacts with RIG-I and TRAF6. (A, B) HEK-293T cells grown in 100-mm dishes were co-transfected with the indicated plasmids encoding the RIG-I (A) or TRAF6 (B) expression vector (4 μg) and HA-USP25WT/ HA-USP25C178A/ HA-USP25H607A (4 μg) using Lipofectamine 2000. Immunoprecipitation (IP) was analyzed by immunoblots (IB) with anti-HA (top panels) and anti-Flag (middle panels). The input tagged proteins were verified with the indicated antibodies. (TIF) [file pone.0080976.s001.tif]
