# Supplementary material for: Ubiquitin-Specific Proteases 25 Negatively Regulates Virus-Induced Type I Interferon Signaling
Source: PLoS One. 2013 Nov 18;8(11):e80976. doi: 10.1371/journal.pone.0080976 (PMC3832446; doi:10.1371/journal.pone.0080976)
Supplement: Table S1 — Sequence for siRNAs of human USP25. (DOC) [file pone.0080976.s002.doc]

**Table S1 Sequence for siRNAs** of human USP25

| RefSeq | symbol | Sequence（5’-3’） | Sequence（3’-5’） |
| --- | --- | --- | --- |
|  | siUSP25-1 | cuaugguuccggucccaaadtdt | uuugggaccggaaccauagdtdt |
| NM_013396 | siUSP25-2 | gaaagauuaccucacgguadtdt | uaccgugagguaaucuuucdtdt |
|  | siUSP25-3 | gacaugaugaagaauugaudtdt | aucaauucuucaucaugucdtdt |
