# Supplementary material for: Ubiquitin-Specific Proteases 25 Negatively Regulates Virus-Induced Type I Interferon Signaling
Source: PLoS One. 2013 Nov 18;8(11):e80976. doi: 10.1371/journal.pone.0080976 (PMC3832446; doi:10.1371/journal.pone.0080976)
Supplement: Table S2 — Primers for effective genes of innate immunity used in real-time RT-PCR. (DOC) [file pone.0080976.s003.doc]

**Table** S2 Primers for effective genes of innate immunity used in real-time RT-PCR

| RefSeq | symbol | Forward primer | Reverse primer |
| --- | --- | --- | --- |
| NM_002176 | IFN-β | tctttccatgagctacaacttgct | gcagtattcaagcctcccattc |
| NM_005101 | ISG15 | gggacctgacggtgaagatg | cgccgatcttctgggtgat |
| NM_00201 | ISG20 | ccgtggccaggctagagat | ccgctcatgtcctctttcagt |
| NM_001547 | ISG54 | cacctctggactggcaatagc | gtcaggattcagccgaatgg |
| NM_001270927 | ISG56 | gctttcaaatcccttccgctat | gccttggcccgttcataat |
| NM_001565 | IP-10 | gtccacgtgttgagatcattgc | cctttccttgctaactgctttca |
| NM_002046 | GAPDH | tcatgaccacagtccatgcc | ggatgaccttgcccacagcc |
